# Adjusting the dose of traditional drugs combined with immunotherapy: reshaping the immune microenvironment in lung cancer

**DOI:** 10.3389/fimmu.2023.1256740

**Published:** 2023-10-09

**Authors:** Linlin Wang, Changqi Du, Bing Jiang, Lin Chen, Zibing Wang

**Affiliations:** ^1^ Department of Immunotherapy, Affiliated Cancer Hospital of Zhengzhou University & Henan Cancer Hospital, Zhengzhou, Henan, China; ^2^ Gansu University of Traditional Chinese Medicine, Lanzhou, Gansu, China; ^3^ Guangzhou Medical University-Guangzhou Institute of Biomedicine and Health (GMU-GIBH) Joint School of Life Sciences, Guangdong-Hong Kong-Macau Joint Laboratory for Cell Fate Regulation and Diseases, Guangzhou Medical University, Guangzhou, Guangdong, China

**Keywords:** lung cancer, immune checkpoint inhibitors, immune microenviroment, drug dose adjustment, immunothearpy

## Abstract

Immunotherapy is currently the most promising clinical treatment for lung cancer, not only revolutionizing second-line therapy but now also approved for first-line treatment. However, its clinical efficiency is not high and not all patients benefit from it. Thus, finding the best combination strategy to expand anti-PD-1/PD-L1-based immunotherapy is now a hot research topic. The conventional use of chemotherapeutic drugs and targeted drugs inevitably leads to resistance, toxic side effects and other problems. Recent research, however, suggests that by adjusting the dosage of drugs and blocking the activation of mutational mechanisms that depend on acquired resistance, it is possible to reduce toxic side effects, activate immune cells, and reshape the immune microenvironment of lung cancer. Here, we discuss the effects of different chemotherapeutic drugs and targeted drugs on the immune microenvironment. We explore the effects of adjusting the dosing sequence and timing, and the mechanisms of such responses, and show how the effectiveness and reliability of combined immunotherapy provide improved treatment outcomes.

## 1 Introduction

According to the International Agency for Research on Cancer’s World Cancer Statistical Report, approximately 1.8 million deaths occur annually due to lung cancer, followed by rectal cancer, liver cancer, and stomach cancer (1). Lung cancer is most prevalent among male patients and ranks second among female patients (2) due to its initial asymptomatic nature and difficulty in detection (3, 4). Currently, lung cancer holds the highest incidence and mortality rate globally (5). Smoking causes 80% of lung cancer deaths, while other risk factors include radon gas, asbestos, cumulative exposure to air pollution, polycyclic aromatic hydrocarbon emissions, and genetic factors (6).

**Figure 1:**
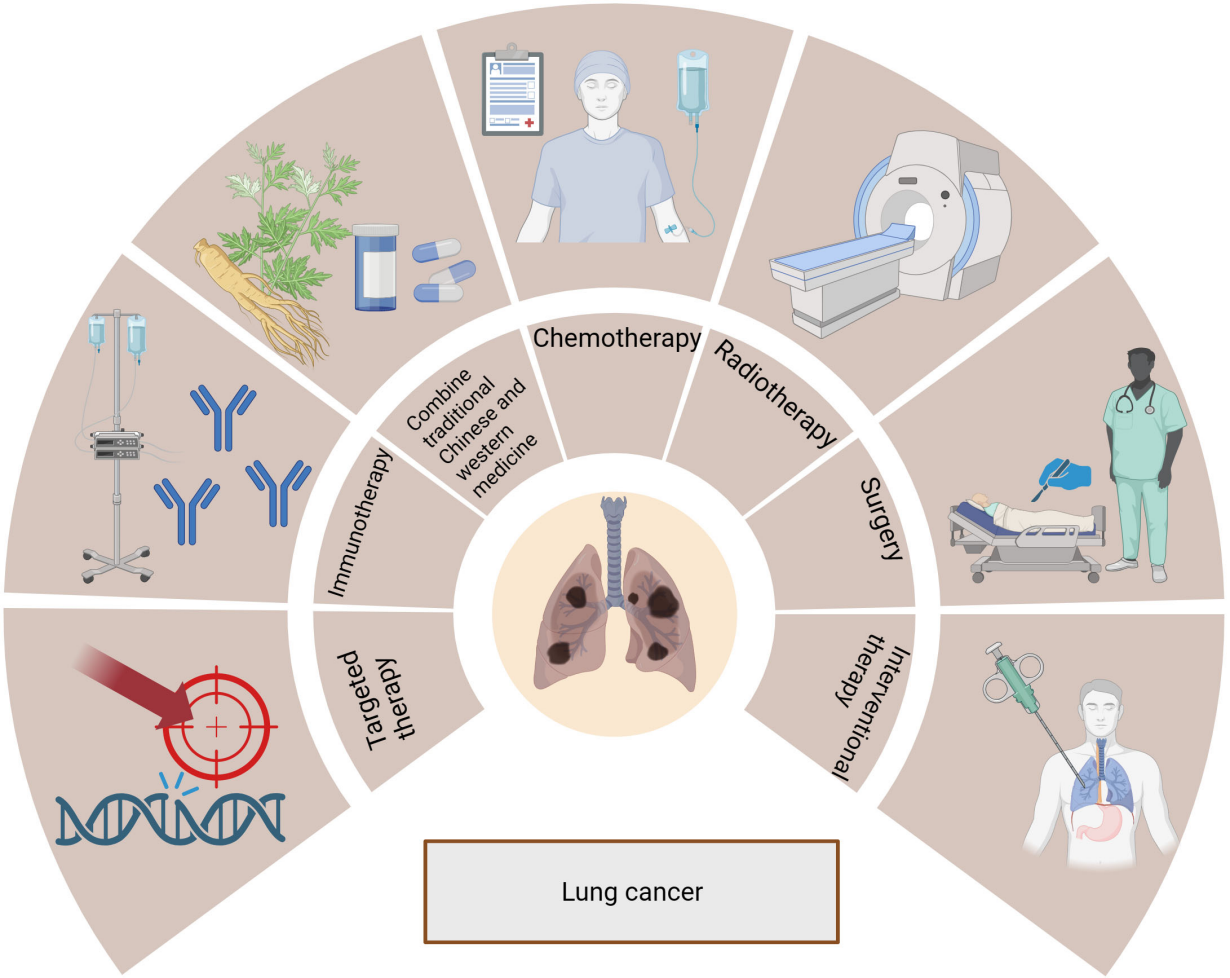
Treatments for lung cancer include surgery, radiotherapy, chemotherapy, targeted therapy, immunotherapy, interventional therapy, and combination of traditional Chinese and Western medicine.

Lung cancer is categorized based on histology into non-small cell lung cancer (NSCLC) and small cell lung cancer (SCLC) (7). NSCLC accounts for 80-85% of all lung cancer cases and includes adenocarcinoma, squamous carcinoma, and other histological subtypes (8). Poor prognosis usually follows an advanced NSCLC diagnosis (9), but new insights into the molecular mechanisms of disease progression and an increased understanding of the disease have allowed for the development of novel treatment options. These treatments include surgery, radiation therapy, chemotherapy, targeted therapy, immunotherapy, interventional therapy, and a combination of Chinese traditional and western medicine, as shown in **Figure 1**. Significantly improved survival rates have been observed in lung cancer patients with the continuous improvement of systemic therapy.

Lung cancer treatment has shifted from indiscriminate cytotoxic chemotherapy to more refined targeted agents. The development of small molecule drugs and monoclonal antibodies that target specific components of dysfunctional molecules or immune pathways, as well as mutated genes that target lung cancer, and the development of more personalized combinations based on different conditions, has led to more optimal treatment options for lung cancer (10). Currently, immunotherapy is the most promising clinical treatment for lung cancer (11, 12). The goal of cancer immunotherapy is to elicit a cellular immune response (6, 13). Immunotherapies for lung cancer include tumor-related vaccines, chimeric antigen receptor (CAR)-T, T cell receptor (TCR)-T cell therapy, tumor infiltrating lymphocytes (TILs) therapy, lysing viruses, targeted antibodies for lung cancer, and immune checkpoint inhibitors (ICIs) (6). Among them, ICIs are the most widely used in clinical practice,which have not only revolutionized second-line treatment, but they are now also approved for first-line treatment (14, 15). Cytotoxic T lymphocyte associated antigen 4 (CTLA-4) (3, 16) is the first antibody in immunotherapy to be approved by the U.S. Food and Drug Administration (17). Since the discovery of CTLA-4, several immune checkpoint proteins have been discovered, including programmed death-1 (PD-1), T-cell immunoglobulin and ITIM domain (TIGIT), T-cell immunoglobulin domain and mucin domain-containing molecule-3 (TIM-3), lymphocyte activation gene 3 (LAG-3), V-domain Ig suppressor of T cell activation, B and T cell lymphocyte attenuator and cluster of differentiation 200. Among the most common clinical treatments for NSCLC are PD-1 monoclonal antibodies, including nivolumab and pembrolizumab (18). However, their clinical effectiveness is not high and not all patients benefit from them. The response rate after second-line treatment with nivolumab is approximately 20%. First-line treatment with pembrolizumab is currently limited to patients with a PD-1 ligand (PD-L1) tumor percentage score above 50%, which accounts for approximately one-third of NSCLC patients, and has an response rate of 69% (15). The upregulation of PD-1 expression in TILs is one of the main immunosuppressive mechanisms in the tumor microenvironment (TME). The TILs interact with ligands (PD-L1 and PD-L2), leading to a decrease in CD8^+^ T cells and an increase in regulatory T cells (Tregs), suppressing the function of CD8^+^ T cells or causing immune escape in response to an adaptive response to interferon (IFN) signaling (17, 19). Other factors that affect the effectiveness of treatment include the absence of tumor expression of MHC class I molecules (20, 21), low numbers of CD4^+^ T cell infiltrates in tumor tissue, high numbers of myeloid-derived suppressor cells (MDSC), and low expression of PD-L1 on cancer cells. Based on these potential mechanisms, the antitumor efficacy of immunotherapy can be boosted with the use of other types of treatment. Thus, finding the best combination strategy to expand anti-PD-1/PD-L1-based immunotherapy is currently a hot issue in lung cancer research (22).

In this paper, we review what is known about the current state of NSCLC research, with a particular emphasis on the TME, the current available drugs for treatment, and the potential for using combined therapies at appropriate doses to improve treatment response while minimizing treatment-related adverse events.

## 2 TME in lung cancer

**Figure 2:**
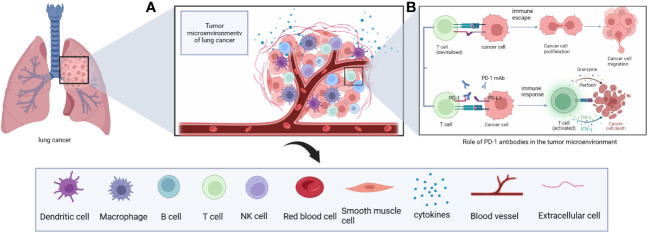
**(A)** The immune microenvironment of lung cancer includes immune cells such as T cells, B cells, NK cells, macrophages, DC cells and cytokines such as IL-2. **(B)** Principles of anti-tumor effects of PD-1 monoclonal antibodies in the immune microenvironment of lung cancer.

Tumor cells and peripheral cells both coexist and compete with each other. Among the surrounding cells are intrinsic and specific immune cells, resulting in a unique environment that varies by tumor type and is highly adapted to tumor behavior; this is referred to as the TME (21). The TME includes tumor-associated macrophages (TAMs), cancer-associated fibroblasts, tissue-specific mesenchymal cells, endothelial cells, intrinsic and specific immune cells, TILs, neutrophils, eosinophils, MDSCs, cytokines and extracellular matrix (23–26) (**Figure 2**). The metabolic status of immune cells in the TME is a key factor affecting their immune response (27) and plays an important role in tumorigenesis, disease progression, and treatment response and prognosis (28). Under normal physiological conditions, innate and acquired immune cells capture and destroy cancer cells through immune surveillance (29, 30). However, in the pathological state, tumor cells can shape the immunosuppressive microenvironment through different mechanisms.

The tumor immune microenvironment consists of a diverse array of cell types, including CD4^+^ T cells, CD8^+^ T cells, B cells, TAMs, natural killer cells, CD1c^+^ myeloid and CD141^+^ myeloid dendritic cells, neutrophils, basophils, eosinophils, and mast cells (31). CD4^+^ T cells have different subsets of cells (TH1, TH2, TH17, and Tregs) that perform specialized immunomodulatory functions and secrete different cytokines to enhance or suppress immune responses (32). Tregs are a functional subpopulation of suppressor T cells that express the transcription factor FOXP3 (33). CD8^+^ T cells are activated to secrete IFN-γ and tumor necrosis factor after cell receptors on their surface destroy tumor cells (34). CD4 CTL and CD8^+^ T cells express granzyme and perforin, which are effectors that mediate cytotoxic activity in target cells (32). The activation process of macrophages is highly plastic, and depending on signals in the TME, macrophages can be polarized into M1 or M2 functional phenotypes (35, 36). M1 macrophages secrete IFN, interleukin (IL), nitric oxide synthase, and reactive oxygen species to exert and enhance anti-tumor immunity (35, 37). M2 macrophages are associated with high expression of IL-10, IL-1β and vascular endothelial growth factor (VEGF) *in vivo*, and form a beneficial survival environment for tumor cells by suppressing immunity and promoting tumor angiogenesis, invasion and distant metastasis (35, 38).

Tumor cells can shape the immunosuppressive microenvironment through nutritional competition, secretion of cytokines, the release of metabolites and modulation of immune cell metabolism to affect immune cell growth, development and differentiation, thereby increasing the function of immune cells toward a pro-tumor phenotype, a process that helps promote the immune escape of tumor cells themselves (27). When PD-L1 on tumor cells binds to PD-1 on T cells, the T cells are unable to specifically recognize the tumor cells, and this also results in immunosuppression (39). If this immunosuppression caused by tumor cells is reversed by drugs, the immune cells can resume their normal function of recognizing and killing tumor cells.

## 3 Low dose of chemotherapy drugs in combination with PD-1/L1 monoclonal antibody

More than 100 chemotherapeutic drugs have entered clinical use since 1948; they are divided into four main categories: 1) alkylating agents such as cyclophosphamide, cisplatin, and oxaliplatin; 2) antimetabolites such as pemetrexed, gemcitabine, and fluorouracil; 3) botanicals such as vincristine and paclitaxel; and 4) antibiotics such as doxorubicin and bleomycin. Chemotherapeutic drugs are thought to produce anti-proliferative or cytotoxic effects during cell division (40), selectively killing cells that are proliferating rapidly in the body. Thus, while killing tumor cells, bone marrow suppression may also occur with a decrease in neutrophils, lymphocytes, platelets and hemoglobin (41). In addition, adverse skin reactions to chemotherapy occur in 30-40% of patients (41). High doses of chemotherapy drugs can also cause significant liver and kidney damage and gastrointestinal complications, side effects that many patients do not tolerate well (42). Thus, chemotherapy combined with immunotherapy seems to be somewhat contradictory because chemotherapy can kill anti-tumor immune cells. To further improve the treatment efficacy in clinical treatment of lung cancer with a combination of chemotherapy drugs and PD-1 monoclonal antibody, the chemotherapy dose may be reduced to reduce the killing effect on immune cells (43).

**Figure 3:**
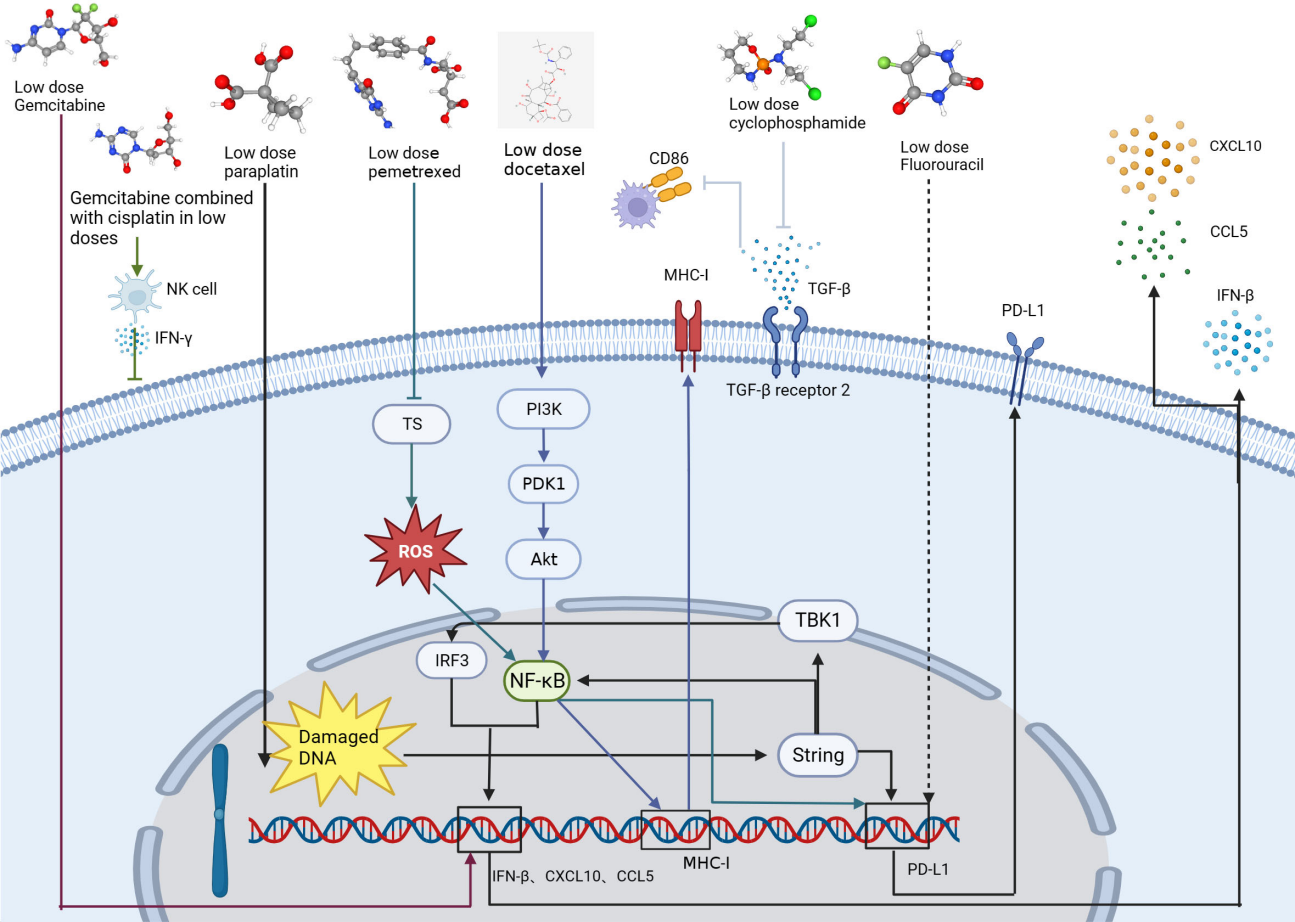
Mechanisms by which low-dose chemotherapeutic agents (gemcitabine, decitabine, carboplatin, pemetrexed, doxorubicin, cyclophosphamide, and fluorouracil) act on tumor cells and enhance antitumor immune function. The arrows of each color represent the pathway by which each drug acts on the lung cancer.

Recent evidence indicates that some chemotherapy drugs at low doses have anti-angiogenic and sometimes even immunomodulatory effects (44). Several new studies have demonstrated that small doses of gemcitabine combined with cisplatin can not only cause immunogenic death of lung cancer tumor cells but can also directly activate NK cells and increase IFN-γ secretion, thus inhibiting tumor growth. The optimal antitumor outcome was observed in *in vivo* experiments when administering a low dose of gemcitabine (30 mg/kg) (45) (**Table 1**, **Figure 3**).

**Table 1:** Modulation mechanisms of the anti-tumor immune effect induced by low-dose chemotherapy.

Drugs	Medication regimen	Adjusted ratio of chemotherapeutic agents (low / standard dose)	Immune cells impacted	Signaling pathway or target	Cytokines	References
Gemcitabine combined with cisplatin in small doses	low-dose (30 mg/kg) gemcitabine	25%	NK cell		IFN-γ, HMGB1	(45)
Low-dose carboplatin	20 mg/kg	26.7%	CD8+ T cell	STING/TBK1/IRF3 STING-NF-κB	CXCL10, CCL5	(42)
Sub-lethal dose of pemetrexed/5-FU	100nM	35.7%	TIL	TS-ROS-NF-κB-PD-L1	IL-2	(46)
Low-dose gemcitabine with SRA737+ anti-PD-L1	gemcitabine (40 mg/kg, 1/7, first day of the week), SRA737+ (100 mg/kg, 2/7, first and second day of the week) and anti-PD-L1 (300 μg, 1/7, third day of the week )	33.3%	CD8+ T cell, DC, M1 macrophage, M2 macrophage, MDSC	STING/TBK1/IRF3	Type 1 IFN, IFN-β, CCL5 and CXCL10	(47)
Low-dose high-density DTX or PTX	DTX (11 mg/kg) or PTX (11 mg/kg)	33.3%	APC, T cell	PI3K/AKT/NF-κB	HMGB-1	(48)
Small doses of cyclophosphamide	25 mg/kg every other day	55.6%	CD4+T cell, CD8+ T cell, Treg		TGF-β	(49)
Rhythm Vincristine combined with Endo	1/10-1/3 of the maximum daily dose	10%~33.3%		HIF-1α and CEPS	CD31, VEGF	(50)

Conventional chemotherapy attempts to maximize efficacy and directly eradicate tumor cells using doses close to the maximum tolerated dose (MTD) and has been the standard of care in lung cancer treatment (51, 52). Even with the use of intensive chemotherapeutic agents, remission rates as well as survival remain poor for lung cancer patients. However, metronomic chemotherapy (MET) is dosed at one-tenth to one-third the MTD (52) and defined as (53) the rhythmic chemotherapy of low-dose cytotoxic drugs with short or no drug-free breaks over prolonged periods (51). MET have shown promising anti-angiogenic properties (53, 54), as well as anti-tumor immune activation, while limiting side effects (54–56). Zhong et al. conducted a study to investigate the effects of three different rhythmic chemotherapy regimens on tumor growth in C57BL mice. The researchers injected 10^6^ cells into the right abdomen of the mice and after four days, administered different doses of cyclophosphamide for a period of three weeks. The three regimens used were MTD, which consisted of three doses of 150 mg/kg during the first week only; Met-1, which was 170 mg/kg given every six days; and Met-2, which was 25 mg/kg given every other day. The results of the study revealed that continuous administration of low-dose cyclophosphamide (Met-2) had a significant impact on the tumor microenvironment. Specifically, there was an increase in the number of CD4^+^ and CD8^+^ T cells, while the number of Tregs decreased. Low doses of cyclophosphamide also decreased the expression of transforming growth factor (TGF-β) receptor 2 by up-regulating the expression of E-calcineurin and down-regulating the expression of N-calcineurin (49) (**Table 1**, **Figure 3**). TGF-β can act as an immunosuppressive factor, and tumors with high expression of TGF-β can escape the surveillance of the immune system and inhibit expression of CD86 by TAMs. As CD86 is a tumor suppressor (57, 58), this implies that inhibition of TGF-β may enhance the antitumor immune response. In one study, co-administration of a Toll-like receptor 9 agonist and TGF-β2 inhibitor not only effectively exerted anti-tumor effects, but also led the TME to have T and NK cell enrichment and improved immunosuppression (59). In addition, sustained regular low-dose cyclophosphamide administration exerts anti-angiogenic effects by inhibiting the expression of VEGF, which can have a sustained tumor suppression effect and has the advantages of less toxic side effects and less drug resistance than conventional MTD administration. A prominent feature of this anti-angiogenic effect is tumor stabilization, not rapid tumor destruction (60).

In an *in vivo* study, rhythmical treatment with vincristine combined with Endo (Met NVB+Endo) gave better results than a maximum tolerated dose of vincristine combined with Endo (MTD NVB+Endo) in terms of anti-tumor responses, reduction of CD31, VEGF, HIF-1α and CEPS expression, as well as reduction of toxic side effects and induction of apoptosis. In this experiment, mice were administered vincristine at the MTD of 10 mg/kg, with the MET ranging from 1/10 to 1/3 of the maximum daily dose. In addition, the combination showed better antitumor effects than either drug alone (50). VEGF expression, detected by western blotting, indicated reduced VEGF expression levels in the Met NVB+Endo group and MTD NVB+Endo group (**Table 1**, **Figure 3**). The potent antitumor effect of MET in combination with anti-angiogenic drugs through enhanced inhibition of tumor-associated angiogenesis is consistent with previous findings (50, 61). Despite the observed positive outcomes in terms of tumor control and reduced side effects, conclusive Phase III trial results are yet to be established. Moreover, patient drug dosage and dosing intervals are currently determined empirically, and inter-individual variances necessitate a criterion for patient categorization (62).

Low-dose chemotherapy drugs combined with ICIs have a synergistic effect in the treatment of tumors (44). Li Zhou et al. (42) showed carboplatin activated the STING/TBK1/IRF3 signaling pathway and the STING-NF-κB signaling pathway, then experimentally verified that low dose of carboplatin could increase PD-L1 expression in lung cancer cells. In addition, a low dose (20 mg/kg) of carboplatin also increased the infiltration of cytotoxic CD8^+^ T cells and the secretion of the chemokines CXCL10 and CCL5 compared to a high dose (75 mg/kg) (42) (**Table 1**, **Figure 3**). Low-dose carboplatin in combination with PD-1 monoclonal antibody significantly improved the anti-tumor effect compared to both PD-1 monoclonal antibody alone and carboplatin alone without significant toxic side effects (42).

Lu et al. (46) found that sublethal doses of pemetrexed (100nM) and 5-fluorouracil (5-FU) could upregulate PD-L1 expression and regulate TIL activity in NSCLC cells, and found that pemetrexed or 5-FU elevated PD-L1 protein levels in a dose-dependent manner. *In vivo* experiments, the combination of pemetrexed (100 mg/kg) with a PD-1/PD-L1 blocker (3 mg/kg) further enhanced the antitumor immune response. The antimetabolite pemetrexed induced PD-L1 upregulation by activating the ROS-NF-κB signaling pathway through inactivation of thymidylate synthase and thus in combination with PD-1 monoclonal antibody activation of CD4^+^ T cells and CD8^+^ T cells provides a more favorable immune microenvironment for tumor growth inhibition (46) (**Table 1**, **Figure 3**).

Sen et al. found that low-dose gemcitabine (40 mg/kg, first day of the week) in combination with SRA737+ (100 mg/kg, first and second day of the week) and anti-PD-L1 (300 μg, third day of the week) combination therapy in the treatment of tumor-bearing mice had significantly better antitumor effects than single-agent or two-by-two dosing regimens. This triple therapy increased T-cell infiltration, decreased T-cell depletion, and significantly increased antigen-presenting cell subpopulations. This was demonstrated by a significant increase in the number of CD8^+^ cytotoxic T cells, dendritic cells, and M1 macrophages and a significant decrease in the number of immunosuppressive M2 macrophages and MDSC. Triple therapy also increased the expression of the type 1 interferon gene, IFN-β, and the chemokines CCL5 and CXCL10 (47) (**Table 1**, **Figure 3**).

He et al. demonstrated that low-dose high-density DTX (11 mg/kg) or PTX (11 mg/kg) indirectly activated the killing effect of T cells on tumor cells by activating the PI3K/AKT/NF-κB signaling pathway, promoting the exposure of antigen on the surface of the tumor cells, and further activating the antigen-presenting function of antigen-presenting cells. Combined with PD-1/PD-L1 monoclonal antibody, it can increase the expression of type 1 macrophages, dendritic cells (DCs) and cytotoxic CD8^+^ T cells (48) (**Table 1**, **Figure 3**).

In summary, low-dose chemotherapy drugs have vascular and immunomodulatory effects, and their combined application with ICIs has a synergistic effect. One of the important tasks ahead is to conduct more research to further determine the optimal dose, frequency, and sequence of chemotherapy drugs that achieve the best antitumor efficacy with ICIs.

## 4 Targeted drugs and lung cancer

Current targeted therapy for lung cancer includes the targets VEGF, EGFR, ALK, ROS1, MET, BRAF, NTRK, RET, and RAF (63). As research continues, many other oncogenic drivers, such as HER2 exon 20 insertion mutations are being identified, and the efficacy data of targeted therapies are constantly being updated (64). Targeted therapies are undoubtedly a milestone in the development of cancer therapy. They play a key role in early disease detection and increase our understanding of tumor evolution and treatment resistance. Targeted therapies represent one of the future directions of precision oncology approaches.

### 4.1 Current status of anti-angiogenic drugs for lung cancer

Angiogenesis involves several growth factors (65), among which the VEGF family consists of VEGF-A, VEGF-B, VEGF-C, VEGF-D and placental growth factor (66). VEGF-A is a major regulator of angiogenesis and is closely associated with angiogenesis in NSCLC, and VEGF receptor (VEGFR)-1 and VEGFR-2 are both receptors for VEGF-A (66, 67). VEGFR-1 binds VEGF-A with a higher affinity than VEGFR-2 (68). Anti-angiogenic drugs normalize local blood vessels (69) and can be divided into four categories: anti-VEGF monoclonal antibodies (mAb), anti-VEGFR mAb, induced VEGF-trap receptors, and VEGFR tyrosine kinase inhibitors (TKIs). In addition, endothelial inhibitors inhibit endothelial cell proliferation by inhibiting a series of angiogenic factors, such as recombinant human vascular endothelial inhibitor (Endo) (66, 69).

Since the approval of the first anti-angiogenic drug, bevacizumab, for the treatment of NSCLC, anti-angiogenic therapy has become a popular strategy for the treatment of advanced NSCLC (66). Bevacizumab is a recombinant humanized monoclonal antibody against VEGF-A, and patients treated with bevacizumab have improved OS. However, the addition of bevacizumab leads to increased toxicity, particularly neutropenia, thrombosis, hypertension, proteinuria, and bleeding events (15). Bevacizumab is not approved for the treatment of NSCLC due to the high risk of bleeding reported in early trials. This risk is associated with the central site of the disease, which often infiltrates the large mediastinal vessels (70). Ramucirumab, a recombinant human IgG1 monoclonal antibody targeting VEGFR-2, also blocks the activation of VEGFR-2 by ligands other than VEGF-A compared to bevacizumab (10, 15, 61, 71). Combination therapy with ramucirumab significantly improves progression free survival and overall survival, but adverse effects are also common (72).

Anti-angiogenic TKIs target VEGFR1-3 in addition to a variety of other kinases (15). The most common, anlotinib, was approved by the National Drug Administration on 8 May 2018 and 30 August 2019 for third-line treatment in patients with advanced NSCLC and SCLC, respectively (73). Despite the wide range of targets of TKIs, most TKIs appear to be only weakly effective in the treatment of NSCLC. Several clinical trials are investigating whether anti-angiogenic drugs can stimulate immunity, improve immunosuppression and thereby enhance antitumor immunity. New therapeutic targets including metabolic intermediates of vascular endothelial cells and cellular components of the TME may lead to the discovery of new novel targets beyond the VEGF family (28).

### 4.2 Immunomodulatory effects of anti-angiogenic drugs

**Figure 4:**
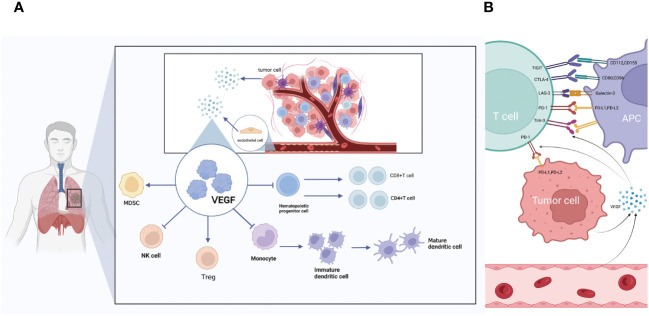
**(A)** The relationship between VEGF and immune cell action. VEGF secreted by tumor cells and endothelial cells can promote immunosuppressive MDSC and Treg cells. Inhibit the differentiation of monocytes into mature DC, inhibit the differentiation of T cells and inhibit NK cells. **(B)** The relationship between VEGF and immune checkpoints. VEGF secreted by tumor cells and endothelial cells can promote multiple immune checkpoints including TIGIT, PD-L1, LAG-3, CTLA-4 and TIM-3.

Clinical studies have shown that VEGF can affect immune cells (74, 75), including inhibiting the differentiation of thymic hematopoietic progenitors to CD8^+^ and CD4^+^ T cells, suppressing the proliferation cytotoxic activity of effector T cells through binding to VEGFR2, reducing the activity of natural killer cells, increasing Tregs and MDSC in the TME (68, 76) and up-regulating a variety of immune checkpoints, such as PD-L1, CTLA-4, TIGIT, TIM-3 and LAG-3 (77, 78) (**Figure 4**).VEGF blockade has been shown to reduce VEGF-mediated inhibition of DC maturation (79) (**Figure 4**),which can be reversed by anti-angiogenic drugs targeting VEGF-A-VEGFR. Given these results, the association of anti-angiogenic molecules with immunomodulatory agents with suppressive checkpoints may be of particular interest in VEGF-A producing tumors. The combination of bevacizumab and atezolizumab reverses the immunosuppression produced by VEGF and lifts PD-L1-mediated immunosuppression (80). In experimental studies, anti-angiogenic agents bevacizumab and sorafenib (polytyrosine kinase VEGFR2 inhibitors) reversed VEGF-mediated inhibition of monocyte differentiation to DCs *in vitro* (81).

In a study involving experimental animals with lung adenocarcinoma, a reduction in the infiltration of CD8^+^ T cells into tumors was observed in the anti-VEGFR2 antibody (DC101) group receiving a low-dose (10 mg/kg). However, there was no notable variation in the percentage of T cells that underwent *in vitro* treatment with a combination of medium-dose (20 mg/kg) and high-dose (40 mg/kg) DC101 and anti-PD1 antibody. Combining low-dose anti-VEGFR2 antibody with anti-PD1 antibody treatment resulted in a delay in tumor growth and extended the survival time of mice afflicted with tumors (82).VEGF-A upregulates both LAYN and immune receptors in human CD8^+^ T cells such as TIGIT (82). LAYN is a key gene in the regulation of immunity, and a bioinformatics analysis showed that LAYN is associated with prognosis and the level of immune infiltration of CD8^+^ T cells, CD4^+^ T cells, macrophages, neutrophils, and DCs in patients with several cancers, especially colon and gastric cancers. In addition, LAYN expression may contribute to the regulation of TAMs, DCs, T-cells, and Tregs in colon and gastric cancers (83). However, no experiments have been performed to verify this conclusion. In a human tissue lymphocyte transcriptional atlas, analyzing RNA-SEQ data from colorectal and NSCLC tumors along with normal colon and lung samples, high expression of Tregs’ cellular signature genes, such as LAYN, MAGEH1 and CCR8, in whole tumor samples was associated with poor prognosis. This finding highlights the specific expression pattern of immune checkpoints and their ligands in tumor-infiltrating Tregs and effector cells and suggests that their functional relevance should be studied directly at the tumor site (84). PD-1 combined with its ligand PD-L1 allows tumor cells to escape recognition by T cells, achieving immune escape and exerting immunosuppressive effects; the combination of LAYN and its ligand has the potential to stimulate Tregs and further suppress effector T cells. Bioinformatic analysis of data is a resource that can generate and validate hypotheses to increase our understanding of tumor-infiltrating Tregs biology and identify immune targets (84).

### 4.3 Anti-angiogenic drugs combined with chemotherapy and immunotherapy exert powerful anti-tumor effects in lung cancer

In NSCLC, VEGF-A is overexpressed, and the progression of NSCLC is closely associated with angiogenesis. The larger the tumor size of NSCLC or the more advanced the tumor, the more likely it is to undergo excessive, abnormal angiogenesis (66). In one study, angiogenesis was found to be more abundant in lung squamous carcinoma than in lung adenocarcinoma (85). In addition, high levels of circulating VEGF-A are associated with poor prognosis in NSCLC, so using circulating levels of VEGF-A expression to predict patient prognosis may be useful (86). Chinnasamy et al. developed anti-VEGFR2 CAR T cells in a mouse model in which T cells cotransduced with anti-VEGFR-2 CAR and IL-12 infiltrated, expanded and were maintained in tumor tissues for longer. The altered immunosuppression of the TME by anti-VEGFR-2 CAR can lead to effective tumor regression. This is effective in mice, but further efficacy and safety assessments are needed for humans (87).

Chemotherapy combined with anti-angiogenesis drugs such as bevacizumab is more effective against NSCLC than chemotherapy alone. In one study (88), median survival in the chemotherapy plus bevacizumab group for NSCLC was 12.3 months compared with 10.3 months in the chemotherapy alone group. Median progression free survival was 6.2 and 4.5 months for the chemotherapy plus bevacizumab group and chemotherapy alone group, respectively, and the effective rates were 35% and 15%, respectively. Clinically significant bleeding rates were 4.4% and 0.7%, respectively. There were 15 treatment-related deaths in the chemotherapy plus bevacizumab group, including 5 deaths from pulmonary hemorrhage (88). Thus, bevacizumab combined with chemotherapy can significantly improve PFS and effectiveness compared to chemotherapy alone, but the former has an increased rate of bleeding.

In a subsequent study, the dosage and duration of drug use was adjusted to see if side effects, such as bleeding could be reduced while retaining effectiveness. The toxicity, biology and antitumor activity of the rhythmic chemotherapy combined with the bevacizumab regimen were investigated by treating a group of 114 patients, 86 of whom were treated with split-dose cisplatin and oral etoposide plus bevacizumab; 28 patients were treated with split-dose cisplatin and oral etoposide. These patients had no significant toxicity or delay associated with toxicity during the chemotherapy course, and no toxic deaths, bleeding, or serious infections occurred (70). Rhythmic chemotherapy is an emerging approach to the treatment of cancer patients based on the long-term use of low-dose cytotoxic drugs (89). This approach allows higher dose intensities of cytotoxic drugs to be achieved compared to conventional chemotherapy regimens, avoiding dangerous blood concentration spikes (90). The reduction of VEGF and IL-17A levels in the tumor tissue of the 86 patients in the combined treatment group was paralleled by an increase in the percentage of peripheral blood central memory T cells, activated CD62L^+^ cytotoxic T cells and the expansion of activated myeloid-derived DCs expressing CD83 and CD80, activating the immune system; this result was ascribed as partly related to the rhythmic method of chemotherapy administration and partly to the maintenance dosing of bevacizumab (70). This suggests changes in the dosage of chemotherapeutic agents can lift immunosuppression and in combination with anti-angiogenic agents can further activate the immune system. Thus, it is hypothesize that adding immunotherapy on this basis for chemotherapy plus anti-angiogenic agents may maximize the effect of each treatment modality and simultaneously circumvent the side effects due to single drug dose requirements, allowing for long-term treatment to improve survival and quality of life.

Chemotherapy aims to inhibit the over-proliferation of cancer cells but may not effectively control two of the most important conditions in the tumor-permissive environment: neo-angiogenesis and tolerogenic immunity. This conjecture was tested in a prospective randomized trial that included patients with advanced, unresectable pancreatic, non-small cell lung, or prostate cancer (91). One group of patients was given standard chemotherapy and served as a control group; the other group was treated with chemotherapy plus an anti-angiogenic and anti-tumor immune-inducing regimen. The latter group had significantly longer survival, lower blood levels of neovascularization and immune tolerance mediators, and higher levels of anti-angiogenic and anti-tumor immune mediators than the control group (91). The anti-angiogenic effect was monitored by detecting VEGF and vasopressor levels, and the anti-tumor immunomodulation was determined by assessing the number and presence of Tregs and DCs. Several antitumor immune induction regimens are possible, including low-dose rhythm cyclophosphamide, high-dose COX-2 inhibitors, granulocyte colony-stimulating factor, sulfhydryl donors, and blood derivatives containing autologous tumor antigens released into the blood from the patient’s tumor. Whether this antitumor immune induction regimen is equally or more effective if replaced with low-dose rhythm chemotherapy plus ICI or immune inducer plus ICI requires additional study. In an *in vivo* trial, triple combination therapy, i.e., radiation combined with PD-L1 monoclonal antibody and anlotinib, was used to improve the tumor microenvironment and to counteract the immunosuppressive effects of radiation on the tumor microenvironment in Lewis lung cancer mice. Compared with radiation-combined immunotherapy, anlotinib was able to promote infiltration of CD8^+^ T cells and M1-type macrophages and reduce the number of MDSCs and M2 macrophages. In addition, IFN-γ and IL-18 levels were higher, while IL-23, IL-13, IL-1β, IL-2, IL-6, and IL-10 levels were significantly lower. Triple combination therapy also promoted the anti-tumor effects of radioimmunotherapy by downregulating the expression of NF-κB, MAPK and AKT signaling pathways (92) (**Table 2**). The anti-angiogenic and immune-activating effects of anlotinib provide a strong theoretical basis for the clinical treatment of lung cancer (92).

**Table 2:** Regulatory mechanisms of anti-tumor immune effects induced by different targeted drugs.

Drug	immune cells impacted	Cytokines	Signaling pathway or target	References
Bevacizumab	DC, CTL Treg	IL-17	PD-L1, TIGIT, LAG-3, TIM-3, CTLA-4	(70, 77, 78)
Anlotinib	CD8+ T cells, M1 macrophages, MDSCs, M2 macrophages	IFN-γ, IL-18, IL-23, IL-13, IL-1β, IL-2, IL-6, IL-10	NF-κB, MAPK and AKT	(92)
EGFR-TKI	CD8+ T cells, DC FOXP3+ Tregs M2 macrophages	IFN-γ, IL-10	STAT3 pathway, PD-L1	(29, 93)
ALK-TKI DNA-PK inhibitors	T cells CD8+ T cell	IFN-γ TGFβ	PD-L1 PD-L1	(94, 95) (96)

## 5 Current status of other targeted drugs for lung cancer and immunomodulatory effects of low doses on lung cancer

With the identification of alterations in the targeted oncogene, advanced lung cancer can be treated with greater precision (64). Targeted agents are increasingly available as a first-line choice of lung cancer treatment and have improved prognosis and reduced toxicity compared to chemotherapy (97). EGFR mutations and ALK fusions are the most common targeted alterations (98, 99). In the analysis of specific cell populations, different TME modifications were detected in EGFR-positive and ALK-positive tumors compared to EGFR/ALK-negative tumors: TME in EGFR-positive tumors had decreased numbers of CD8^+^ T cells; TME in ALK-positive cases had increased numbers of Tregs. This suggests that in the development of lung cancer different immune cell responses occur (98, 100). In addition, targeted oncogenic alterations were also associated with PD-L1 expression, and upregulated by activation of MAPK, PI3K-AKT-mTOR and JAK-STAT3 signaling pathways in NSCLC cells with altered KRAS, EGFR and ALK activating genes or PD-L1 expression (19, 101–106).

### 5.1 EGFR

EGFR is one of the most common mutation driver genes and is considered an oncogenic factor (107). As a representative of precision medicine, EGFR-TKI therapy significantly alleviates the development of activating mutant EGFR-driven NSCLC (108). EGFR-TKI drugs include erlotinib, afatinib, gefitinib, and osimertinib (63). Madeddu et al. (29) found that EGFR-TKI enhanced MHC class I and class II antigen presentation in response to IFN-γ, increased CD8^+^ T cell and DC levels, eliminated FOXP3^+^ Tregs, inhibited proliferation and differentiation of M2 macrophages, and decreased PD-L1 expression. In another clinical trial of EGFR-TKI combined with ICI therapy, the use of an EGFR-TKI afatinib inhibited CD8^+^ T cell proliferation and a time-related modulation of CD8^+^ T cell proliferation was found in NSCLC patients who received afatinib-targeted therapy for up to 48 weeks during treatment. In the early phase of treatment, afatinib inhibited CD8^+^ T cell proliferation, and in the late phase of treatment, CD8^+^ T cells responded adaptively to afatinib treatment (93). In contrast, the results of several clinical trials of EGFR-TKI combination immunotherapy showed no additional benefit in the treatment of lung cancer (69, 72, 97, 109). The different results of these studies may be related to the different types, specificity and doses used of the EGFR-TKI drugs.

### 5.2 ALK

Immunogenic cell death (ICD) was originally discovered in the context of chemotherapy, but only a small fraction of chemotherapeutic agents can trigger ICD, which is related to their clinical long-term efficacy against cancer and their ability to inhibit DNA-to-RNA transcription (94). In one study, small doses of ALK inhibitors, crizotinib (≤5 μM) and ceritinib, induced ICD when ALK was activated due to chromosomal translocations, suggesting a targeting effect to promote ICD (94) (**Table 2**). In a co-culture system of tumor cells and DC-cytokine-induced killer cells, PD-L1 expression in NSCLC cell lines was associated with EGFR mutations and ALK fusion genes, and ALK fusion protein overexpression increased PD-L1 expression (80). In contrast, Mu et al. (95) found that ALK fusion proteins downregulated PD-L1 expression (**Table 2**). No synergistic effect of the combination of ALK-TKI and PD-1 monoclonal antibody against tumor cells was observed using *in vivo* experiments. One possible explanation is that in ALK-positive NSCLC, ALK-TKI may have a similar role in disrupting PD-1/PD-L1 interactions as anti-PD-1 antibodies, but no additional role. The trial was conducted at the cellular level only, and further *in vivo* experiments are needed to explore the results more accurately. However, the use of crizotinib in combination with cisplatin, followed by PD-1 monoclonal antibody, not only induced ICD but also greatly improved the cure rate in TC1 lung cancer mice (110). There are few studies on ALK-TKI combined with immunotherapy, and results of the studies available to date are not yet convincing.

### 5.3 DNA-dependent protein kinase

Radiation therapy is commonly used in the treatment of lung cancer, but the development of radiation combination therapy is still limited (111, 112). The anti-tumor activity of radiation therapy is mainly derived from the production of double-strand breaks (DSB) in DNA, which, if not repaired, can induce cancer cell death through a variety of mechanisms. Therefore, targeting DSB repair mechanisms in tumors might optimize the effect of radiotherapy. Peposertib (also known as M3814) is a potent and selective DNA-PK inhibitor that effectively inhibits the repair of radiation-induced DNA DSBs, which largely enhances the efficacy of radiotherapy (96). In addition, DNA-PK inhibitor substantially enhanced PD-L1 expression in irradiated cancer cells, providing a clear rationale for combination with PD-L1 targeted immunotherapy (96). Given its critical function, DNA-PK has been targeted in cancer therapy in concert with DNA-damaging agents (113, 114). In addition, DNA-PK inhibitors significantly enhanced the secretion of TGF-β in the tumor microenvironment and the expression of PD-L1 in irradiated cancer cells, providing a theoretical basis for the combination of DNA-PK inhibitors with immunotherapy. Moreover, the addition of M3814 resulted in a significant enhancement of activity in the less immunogenic B16F10 and immune-excluded 4T1 mouse models, a result that was correlated with increased CD8^+^ T cell infiltration in tumors in addition to increased TGF-β secretion as well as PD-L1 expression. In addition to melanoma as well as breast cancer, in lung cancer it has been shown that M3814 alone or in combination with cisplatin enhances the efficacy of anti-PD-L1 monoclonal antibodies (115). However, the clinical use of DNA-PK inhibitors has not been well studied, and there are some unresolved issues, such as short serum half-life due to metabolic instability and unclear optimal doses for combination with immunotherapy.

## 6 Discussion

Immunotherapy is a complex and intriguing area of cancer research (116). How to optimize it is currently a hot topic in cancer research, including how to further improve response rates, expand the population that can benefit, and reduce the incidence of treatment-related adverse events (92). Increasingly, studies indicate that reducing the dose of chemotherapy and adjusting the dosing regimen can not only lead to better anti-tumor effects but can also reduce drug toxicities and regulate the immune microenvironment by modifying the number of immune cells and cytokines to further achieve anti-tumor immunity. Chemotherapeutic drugs can upregulate PD-L1 expression to create favorable conditions for combining PD-1/PD-L1 monoclonal antibodies and allow low-dose chemotherapy combined with immunotherapy to work better. Currently, low-dose chemotherapy combined with immunotherapy has been validated in both *in vitro* and *in vivo* experiments, but clinical studies are limited to date, and more research is needed. Similarly, reducing the dose of anti-angiogenic drugs and adjusting the dosing regimen can improve the anti-tumor effect and activate immunity, which has also been demonstrated in some animal studies. Low-dose anti-angiogenic agents are able to modulate multiple immune checkpoints other than PD-L1, such as TIGIT and LAYN, but all lack substantial experimental data to support this. In a clinical study, the trinity of low-dose chemotherapy plus anti-angiogenic plus immune inducer was found to be more effective than the combination of any two drugs or a single drug (91). For targeting oncogenes, the most common are EGFR mutations and ALK fusions. Studies have shown that normal doses or high doses of EGFR-TKI and ALK-TKI can alter the immune microenvironment in lung cancer, but whether immune activation or immune suppression occurs needs to be further explored.

In conclusion, for the long-term treatment of lung cancer patients, an appropriate dose and targeted combination and dosing regimen based on individual patient differences and tolerance to the drug that provides the best combination strategy to expand anti-PD-1/PD-L1-based immunotherapy can greatly improve the prognosis and quality of life for a patient.

## Author contributions

LW: Writing – original draft, Writing – review & editing. ZW: Writing – review & editing. LC: Writing – review & editing. CD: Writing – review & editing BJ: Supervision, Writing – review & editing.
